# Identification of a prognostic signature and ENTR1 as a prognostic biomarker for colorectal mucinous adenocarcinoma

**DOI:** 10.3389/fonc.2023.1061785

**Published:** 2023-04-27

**Authors:** An Huang, Jingyi Shi, Zhuang Sun, Yong Yang, Zhaoya Gao, Jin Gu

**Affiliations:** ^1^ Key Laboratory of Carcinogenesis and Translational Research (Ministry of Education/Beijing), Department of Gastrointestinal Surgery III, Peking University Cancer Hospital & Institute, Beijing, China; ^2^ Department of Gastrointestinal Surgery, Peking University Shougang Hospital, Beijing, China; ^3^ Department of General Surgery, Peking University First Hospital, Beijing, China; ^4^ Peking Tsinghua Center for Life Science, Peking University International Cancer Center, Beijing, China

**Keywords:** ENTR1, mucinous adenocarcinoma, colorectal cancer, biomarker, prognosis

## Abstract

**Background:**

Mucinous adenocarcinoma (MAC) is a unique clinicopathological colorectal cancer (CRC) type that has been recognized as a separate entity from non-mucinous adenocarcinoma (NMAC), with distinct clinical, pathologic, and molecular characteristics. We aimed to construct prognostic signatures and identifying candidate biomarkers for patients with MAC.

**Methods:**

Differential expression analysis, weighted correlation network analysis (WGCNA), and least absolute shrinkage and selection operator (LASSO)-Cox regression model were used to identify hub genes and construct a prognostic signature based on RNA sequencing data from TCGA datasets. The Kaplan-Meier survival curve, gene set enrichment analysis (GSEA), cell stemness, and immune infiltration were analyzed. Biomarker expression in MAC and corresponding normal tissues from patients operated in 2020 was validated using immunohistochemistry.

**Results:**

We constructed a prognostic signature based on ten hub genes. Patients in the high-risk group had significantly worse overall survival (OS) than patients in the low-risk group (p < 0.0001). We also found that ENTR1 was closely associated with OS (p = 0.016). ENTR1 expression was significantly positively correlated with cell stemness of MAC (p < 0.0001) and CD8+ T cell infiltration (p = 0.01), whereas it was negatively associated with stromal scores (p = 0.03). Finally, the higher expression of ENTR1 in MAC tissues than in normal tissues was validated.

**Conclusion:**

We established the first MAC prognostic signature, and determined that ENTR1 could serve as a prognostic marker for MAC.

## 1 Introduction

Cancer is the second most common cause of disease related disability, years of life lost, and mortality, after cardiovascular diseases, which places a great burden on people worldwide (1). Colorectal cancer (CRC) ranks third and second concerning incidence (10%) and mortality (9.4%) among all cancer types, respectively. The World Health Organization (WHO) estimated that more than 1.9 million new CRC cases and 935,000 CRC-related deaths occurred in 2020 (2). CRC can be divided into adenocarcinoma, adenosquamous carcinoma, squamous cell carcinoma, spindle cell carcinoma, undifferentiated carcinoma and other special types according to histopathology (3). Adenocarcinoma including tubular adenocarcinoma, papillary adenocarcinoma, serrated adenocarcinoma, micropapillary adenocarcinoma, medullary carcinoma, sieve like acne adenocarcinoma, mucinous adenocarcinoma (MAC) and signet ring cell carcinoma, accounts for more than 95% of CRC (4). MAC is characterized by extracellular mucinous components that comprise at least 50% of the tumor tissue, representing a unique clinicopathological CRC type. The proportion of MAC in CRC ranges from 3.9% in Asia, to 10–13.6% in Europe and America (5). Although the prognosis of MAC remains controversial (6–8), a meta-analysis of 34 studies found that mucinous differentiation contributed to a 2-8% increased risk of death (9). In addition, the genetic origin and molecular characteristics of MAC are quite different from those of non-mucinous adenocarcinoma (NMAC) (10). With higher frequencies of *KRAS* and *BRAF* gene mutations, microsatellite instability-high (MSI-H), and CpG island methylator phenotype of high degree (CIMP-H), MAC exhibits clinical features inconsistent with NMAC, where comparatively, MAC is characterized by frequent occurrence in a more advanced stage and at the proximal colon, with a higher rate of peritoneal metastases. Current guidelines for treating MAC are consistent with those for NMAC, which are primarily based on TNM staging and biomarkers, including RAS, BRAF, and microsatellite status. However, there are no treatment recommendations based on the unique features of MAC. Identifying candidate biomarkers and their pathways related to prognosis may aid in understanding the occurrence and progression of MAC, to facilitate individualized treatment for patients.

Tumor biomarkers play a vital role in guiding clinical decision-making with multiple utilization including molecular subtype classification, diagnosis, and prediction of prognosis. Emerging biomarkers, such as mast cells, miRNA, *KRAS* and *BRAF*, may be able to stratify patients with CRC and guide individualized precision treatment (11). The high frequency of *KRAS* and *BRAF* mutations in patients with MAC may be an indicator of poor response to anti-EGFR therapy (12). However, the efficacy of *KRAS*, *BRAF* and biomarkers such as carcinoembryonic antigen and carbohydrate antigen 19-9, which are currently used in clinical practice, is limited in predicting response to treatment and prognosis (13, 14). Although it is urgently needed for prognostic stratification and individualized treatment of patients with MAC, there are few studies focusing on prognostic biomarkers for MAC and progression of MAC, to facilitate individualized treatment for patients. Wang et al. found that PINCH and RAD50 were prognostic biomarkers for MAC (15). However, they did not perform validation or elaborate on the mechanisms involved. Gene expression based analysis is widely valued for its ability to identify potential biomarkers. Weighted correlation network analysis (WGCNA) is a systems biology method used to describe gene association patterns among different samples. It can be used to identify highly covarying gene sets and candidate biomarkers based on the interconnectedness of gene sets and the association between gene sets and phenotypes (16). In this study, we used WGCNA to perform an integrated analysis of RNA-seq and clinical data from The Cancer Genome Atlas (TCGA) database to identify prognostic biomarkers for MAC.

## 2 Materials and methods

### 2.1 Gene expression datasets

We downloaded the RNA sequencing data from TCGA database (https://portal.gdc.cancer.gov/) containing 80 colorectal MAC tissues and 51 normal colorectal tissues with clinical data.

### 2.2 Identification of significant differentially expressed genes in MAC tissues

For the RNA sequencing data we obtained, the R package limma (version 3.40.6) was used to conduct differential expression analysis to identify significant DEGs between MAC and normal tissues. A false discovery rate (FDR) < 0.05 and |log(fold change)| ≥ 2 were used to select DEGs.

### 2.3 KEGG and GO functional enrichment analysis

Enrichment analyses of Kyoto Encyclopedia of Genes and Genomes (KEGG) and Gene Ontology (GO) were conducted by the R software package clusterProfiler (version 3.14.3) to assess the results of the gene set enrichment (17). FDR < 0.01 was taken as statistically significant. The KEGG rest API (https://www.kegg.jp/kegg/rest/keggapi.html) was utilized to gain the latest gene annotations of the KEGG pathway. Genes from the R package org.Hs.eg.db (version 3.1.0) were utilized for GO annotation.

### 2.4 WGCNA and acquisition of hub genes

The TCGA gene expression profile was used to remove genes with a standard deviation of zero in each sample, and goodSamplesGenes of the R software package, WGCNA, was applied to remove outliers and samples (16). WGCNA was then performed to build a scale-free co-expression network. Gene significance was determined as the mediating p-value for each gene (gene significance = lgP) in the linear regression linking gene expression and clinical characteristics. Module eigengenes were determined as the first principal component of each gene module, and the expression of module eigengenes was regarded to represent all genes in a particular module. Module membership was determined as the association between module eigengene and a given gene expression profile. Hub genes bound to MAC were obtained by computing the module membership and gene significance values. The cut-off criteria were |module membership| > 0.8 and |gene significance| > 0.1.

### 2.5 Construction of the prognostic signature

The least absolute shrinkage and selection operator (LASSO) regression model provides a new variable-screening algorithm that can effectively solve the collinearity problem. In our study, LASSO was performed to eliminate redundant factors and to identify the most significant survival-associated hub genes. After excluding patients with a follow-up period of less than 30 days, the R software package glmnet was applied to integrate gene expression data, survival status, and survival time, and the LASSO-Cox method was used for regression analysis. Moreover, 5-fold cross validation was set up to obtain the optimal model.

### 2.6 Validity assessment of the prognostic signature

Patients were classified into two groups depending on the risk scores obtained by the Cox proportional hazards model, and the prognostic difference of the two groups was analyzed by the survfit function of the R software package (18). The Kaplan-Meier survival curve and log-rank test was utilized to assess the statistical significance of the prognosis among the high-risk and low-risk groups. Then, we performed receiver operating characteristic (ROC) analysis at 1, 3, and 5 years using Proc (version 1.17.0.1) of the R software, and evaluated the area under curve (AUC) and confidence intervals using the ci function of Proc.

### 2.7 Establishment of a nomogram

To visualize the prediction of prognosis in patients with MAC, we constructed a nomogram depending on a couple of clinicopathological factors (survival time, survival status, age, sex, T stage, N stage, and M stage) and the 10 genes-based signature using the rms package in R. Harrell’s concordance index (C-index) and calibration curves, which could evaluate the consistency from the predicted survival probability and real observed rates, were conducted to assess the performance of the nomogram.

### 2.8 Clinical significance of the hub genes

We classified the patients into a high-expression group (≥ 50%) and low-expression group (< 50%) depending on the expression levels of the hub genes. The Kaplan-Meier survival curve and ROC analyses were conducted, as previously described. Wilcoxon and Kruskal-Wallis tests were used to analyze the association between the hub gene expression and clinical characteristics.

### 2.9 Gene set enrichment analysis

GSEA software (version 3.0) was utilized to performed GSEA. The c2.cp.kegg.v7.4.symbols.gmt subset of the Molecular Signatures Database (http://www.gsea-msigdb.org/gsea/downloads.jsp) was downloaded to assess the related pathways and molecular mechanisms. FDR < 0.05 was regarded as statistically significant.

### 2.10 Analysis of the relationship of ENTR1 with cell stemness of MAC and immune infiltration

The RNA based stemness scores (RNAss) were calculated using the mRNA signature for each sample according to the algorithm of Malta et al. (19). The relationship between the expression of ENTR1 and infiltration of tumor-infiltrating immune cells was calculated using the R package IOBR (20) with the CIBERSORT (21) and ESTIMATE (22) methods. Correlation analysis was performed using Pearson’s correlation coefficient. Wilcoxon test was used to explore the relationship between gene expression and tumor-infiltrating immune cells.

### 2.11 Immunohistochemistry

The tumor tissues and paired normal tissues from the same patient were surgically harvested at Peking University Shougang Hospital, in accordance with institution-approved protocols. Tissues were collected from 13 patients with CRC, confirmed pathologically as MAC, who underwent radical surgery between January 1, 2020 to December 31, 2020. Informed consent was obtained from all participants involved in the study.

Tissues were fixed in formalin before embedding in paraffin. Paraffin-embedded tissues were sectioned, dewaxed, and dehydrated. Sections were 4 µm in thickness and deparaffinized in xylene, rehydrated in graded ethanol, and washed with TBS (1:20 dilution of 20x TBS, Solarbio, No. T1080) containing 0.3% Triton X-100 (T8200; Solarbio) (TBST) thrice. Sections were pretreated for antigen retrieval using citrate buffer (pH 6.0), cooled to room temperature (RT), and rinsed thrice with TBST. After blocking with 10% goat serum (ZSGB-BIO, ZLI-9021) for 1 h at RT, the tissue slides were incubated at 4°C for 8 h with ENTR1 antibody (Atlas Antibodies, A104721, 1:200 dilution). The sections were washed by TBST thrice after being rewarmed to RT, incubated with 3% H_2_O_2_ for 15 min, and then incubated with goat anti-rabbit IgG (Abcam, ab6721, 1:1000 dilution) at RT for 2 h. A DAB Staining Solution Kit (Gene Tech, Shanghai, GK600705) was used to stain the sections. The sections were counterstained with hematoxylin. Finally, all tissue slides were imaged and assessed using the IHC Profiler plugin (23), based on ImageJ bundled with Java 1.8.0 172 software (24). IHC Profiler used the average gray value (staining intensity) and positive area percentage (staining area) of positive cells as IHC measurement indices, and finally gave the sections three grades of scores as positive (≥2+), low positive (1+), and negative (0). The sections were then evaluated by a pathologist blinded to the nature of the samples, and manual correction was performed for all assessment results. Each tissue sample was replicated thrice. Negative controls without ENTR1 antibody were set for each section.

### 2.12 Statistical analysis

SPSS 26.0 (SPSS, Inc., Chicago, IL, USA) and GraphPad Prism 8 (GraphPad, Inc., CA, USA) were used for analysis. The difference between the two groups was calculated using the paired two-tailed Student’s t-test or Mann–Whitney–Wilcoxon test. Statistical significance was set at *p* < 0.05.

## 3 Results

### 3.1 Identification of the differentially expressed genes of MAC

**Figure 1:**
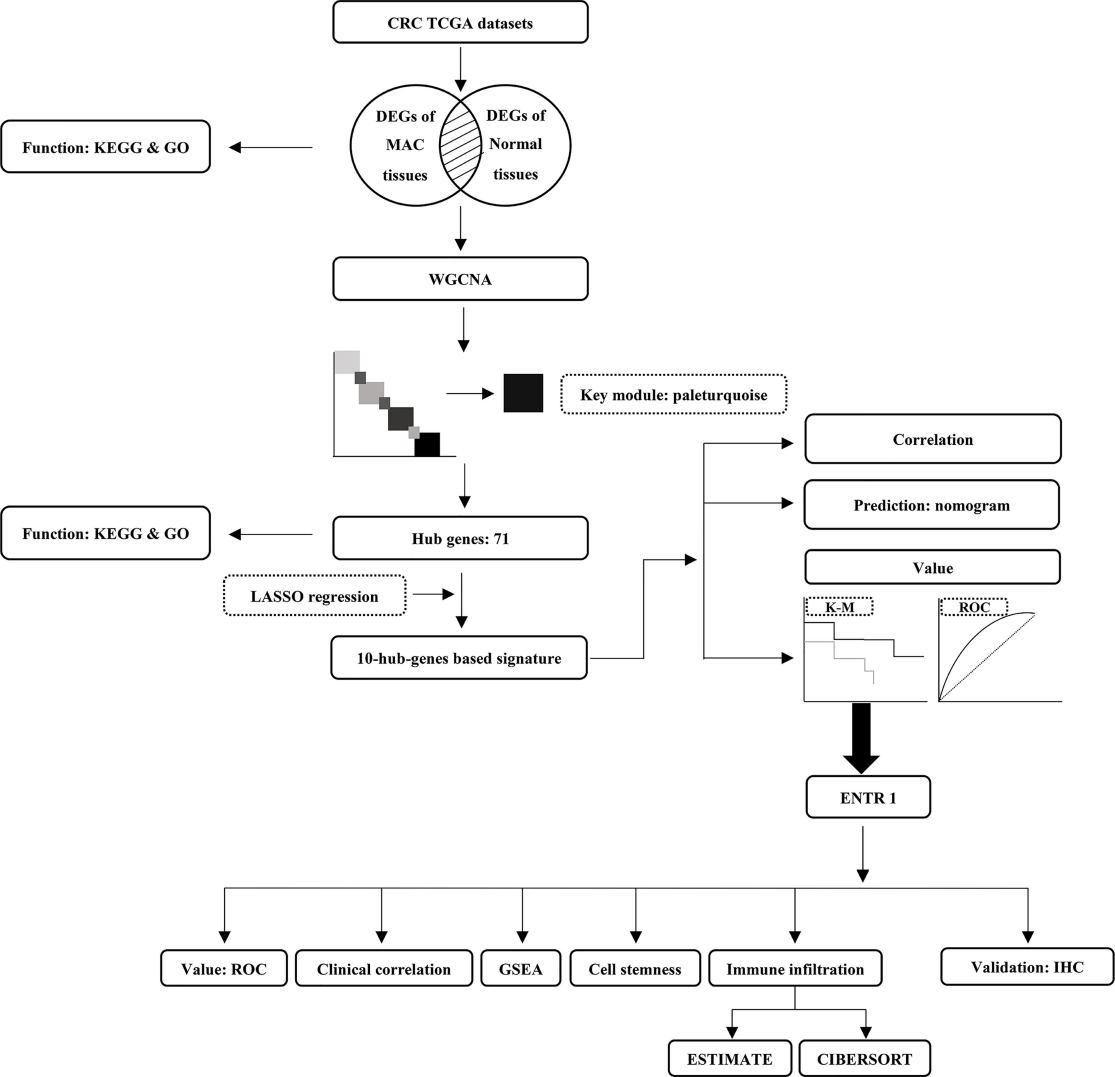
The analysis outline followed in this study. CRC, colorectal cancer; TCGA, The Cancer Genome Atlas; DEGs, differentially expressed genes; MAC, mucinous adenocarcinoma; KEGG, Kyoto Encyclopedia of Genes and Genomes; GO, Gene Ontology; WGCNA, weighted correlation network analysis; LASSO, least absolute shrinkage and selection operator; K-M, Kaplan-Meier survival curve; ROC, receiver operating characteristic; GSEA, gene set enrichment analysis; IHC, immunohistochemistry.

**Figure 2:**
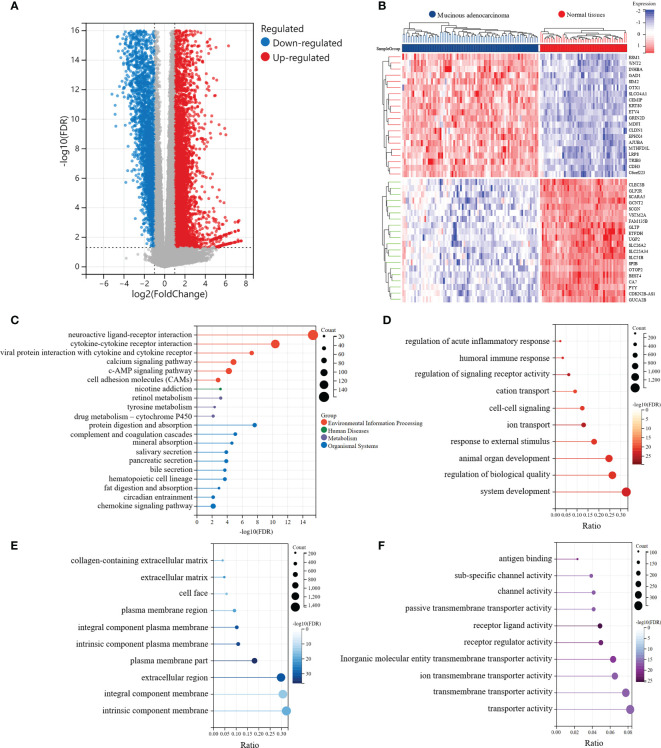
Identification of the significantly differentially expressed genes (DEGs) between MAC and normal tissues of TCGA data. **(A)** Volcano plot of the differential expression and distribution of the TCGA RNA sequencing data between mucinous adenocarcinoma (MAC) and normal tissues. **(B)** Heatmap of the top 20 up-regulated and down-regulated genes in MAC, compared with normal tissues. **(C-F)** Kyoto Encyclopedia of Genes and Genomes (KEGG) and Gene Ontology (GO) functional enrichment analysis of the DEGs (FDR < 0.01). **(C)** The functions of the DEGs in KEGG pathways. **(D)** Top 10 pathways of the DEGs involved in GO biological process (BP) terms. **(E)** Top 10 pathways of the DEGs involved in GO cellular component (CC) terms. **(F)** Top 10 pathways of the DEGs involved in GO molecular function (MF) terms.

The analysis outline followed in this study was displayed in **Figure 1**. We extracted the RNA sequencing data of MAC and normal colorectal samples from the TCGA-COAD and TCGA-READ datasets, and identified DEGs between MAC and normal tissues. Characteristics of the MAC and normal tissues were shown in **Supplementary Table S1**. There were no significant differences between the two groups in age, sex and tissue location. Compared with the normal colorectal tissues, a total of 6,876 genes were upregulated and 3,455 genes were downregulated in MAC tissues (|fold change| ≥ 2, FDR < 0.05). Volcano plot of these genes is shown in **Figure 2A**. **Figure 2B** displays the top 20 genes up- and down-regulated in MAC.

Subsequently, we conducted KEGG and GO enrichment analyses of DEGs. The top five pathways involved in the DEGs, revealed by KEGG enrichment analysis, were neuroactive ligand-receptor interaction (FDR = 4.59e-16), cytokine-cytokine receptor interaction (FDR = 4.35e-11), protein digestion and absorption (FDR = 2.36e-8), viral protein interaction with cytokines and cytokine receptors (FDR = 5.77e-8), and complement and coagulation cascades (FDR = 8.52e-6) (**Figure 2C**). We also explored three main categories of GO enrichment: biological process (BP), cellular component (CC), and molecular function (MF). The top five pathways in the BP category were ion transport (FDR = 2.04e-26), regulation of signaling receptor activity (FDR = 2.04e-26), system development (FDR = 6.42e-24), humoral immune response (FDR = 6.24e-23), and animal organ development (FDR = 1.23e-22) (**Figure 2D**). In the CC category, the top five pathways identified were plasma membrane (FDR = 3.53e-37), intrinsic component of plasma membrane (FDR = 6.29e-34), integral component of plasma membrane (FDR = 3.44e-32), extracellular region (FDR = 8.69e-32), and extracellular matrix (FDR = 8.19e-23) (**Figure 2E**). In the MF category, the top five pathways involved in the DEGs were receptor ligand activity (FDR = 2.81e-26), receptor regulator activity (FDR = 1.13e-23), antigen binding (FDR = 7.95e-22), inorganic molecular entity transmembrane transporter activity (FDR = 2.13e-20), and channel activity (FDR = 1.08e-17) (**Figure 2F**).

### 3.2 WGCNA and identification of hub genes

**Figure 3:**
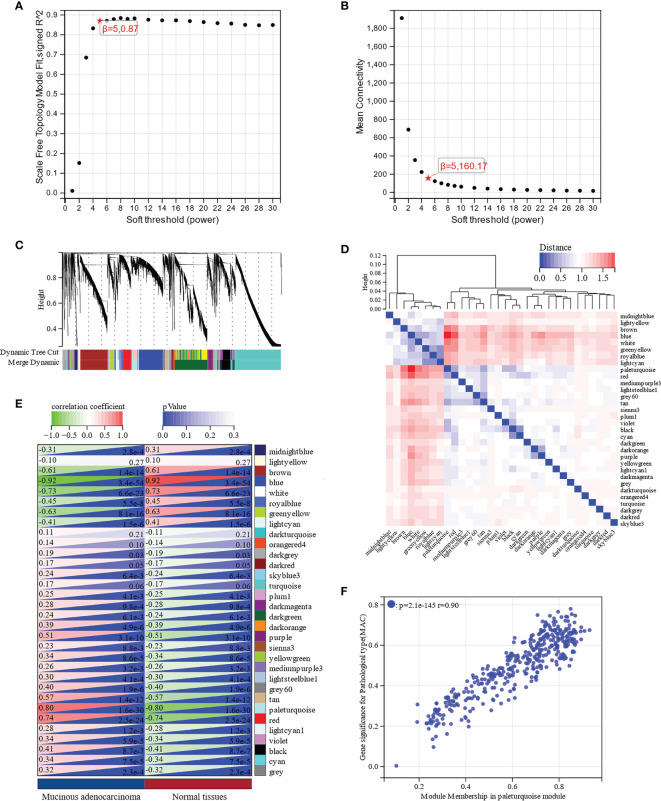
Identification of key modules associated with MAC through Weighted Gene Co-Expression Network Analysis (WGCNA). **(A)** Scale free topology model fit analysis for various soft-thresholding powers. **(B)** Mean connectivity analysis for various soft-thresholding powers. **(C)** Module dendrogram of all differentially expressed genes (DEGs) clustered based on a dissimilarity measure. **(D)** Clustering of module eigengenes and a heatmap of adjacent eigengenes. **(E)** Heatmap of the correlation between module eigengenes and MAC. Each cell contains the correlation coefficient and *p* value. **(F)** Scatter plot of module membership in paleturquoise module and gene significance for MAC.

To determine the crucial modules most correlated with MAC, we performed WGCNA on the DEGs in MAC and normal tissues. Taking a soft-thresholding power of 5 (scale-free R^2^ = 0.87), we identified 32 gene modules (**Figures 3A–D**). From the correlation heatmap of the module eigengenes and MAC, we noticed that the paleturquoise module had the most positively correlation with MAC (*p* = 5.2e-10; **Figure 3E**). The scatter plot of module membership in the paleturquoise module and the gene significance for MAC indicated a high positive correlation (correlation coefficient = 0.90, *p* = 2.1e-145, **Figure 3F**). By setting up the cut-off criteria (|module membership| > 0.8 and |gene significance| > 0.1), we identified 71 hub genes from the 391 genes in the paleturquoise module (**Supplementary Table S2**).

**Figure 4:**
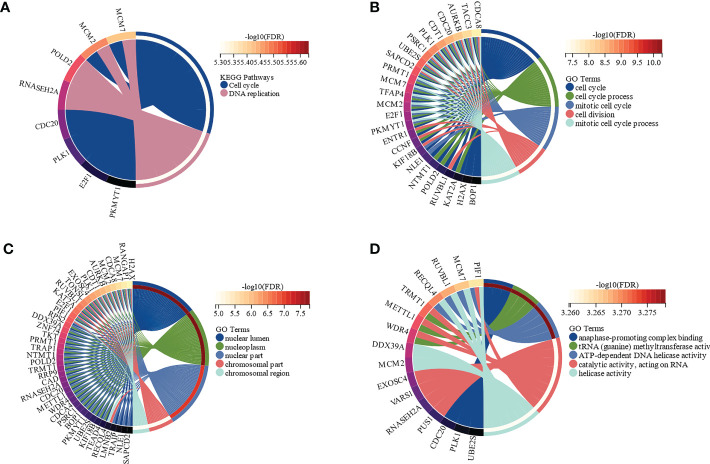
Functional enrichment analysis of the hub genes by Kyoto Encyclopedia of Genes and Genomes (KEGG) and Gene Ontology (GO) (FDR < 0.01). **(A)** The functions of the hub genes in KEGG pathways. **(B)** Top 5 pathways of the hub genes involved in GO biological process (BP) terms. **(C)** Top 5 pathways of the hub genes involved in GO cellular component (CC) terms. **(D)** Top 5 pathways of the hub genes involved in GO molecular function (MF) terms.

The functions and signaling pathways of the hub genes were analyzed using KEGG pathway analysis and GO functional enrichment analysis. According to KEGG pathway analysis, these hub genes were enriched in the cell cycle (FDR = 1.35e-4) and DNA replication (FDR = 1.46e-4) pathways, when FDR < 0.01 was considered statistically significant (**Figure 4A**). GO functional enrichment analysis showed that the highest enriched GO terms in the BP, CC, and MF categories were cell cycle (FDR = 8.15e-8, **Figure 4B**), nuclear lumen (FDR = 1.67e-8, **Figure 4C**), and anaphase-promoting complex binding (FDR = 5.27e-4, **Figure 4D**), respectively. In brief, these results indicated that hub genes were mainly involved in the cell cycle process.

### 3.3 Development and validation of a prognostic model based on the hub genes in patients with MAC

**Figure 5:**
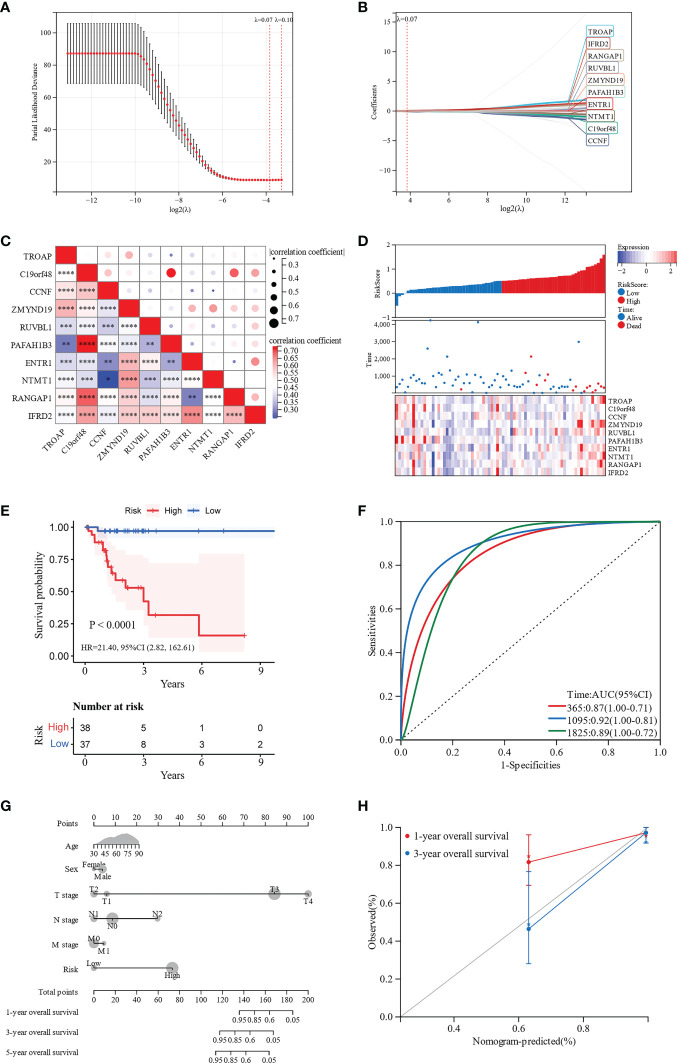
Development and validation of a 10 hub genes-based signature and a nomogram for prognostic prediction. **(A, B)** LASSO regression analysis was performed to develop the prognostic signature. **(C)** Correlation heatmap of the 10 hub genes. **(D)** The distribution of risk scores in patients with MAC. **(E)** Kaplan-Meier survival curves for the low-risk and high-risk groups. **(F)** ROC curves for the 10-genes signature. **(G)** Nomogram for predicting 1-, 3-, and 5-year OS of patients with MAC. **(H)** Calibration curve for the nomogram predicting 1- and 3-year OS with the ideal model. *p < 0.05. **p < 0.01. ***p < 0.001. ****p < 0.0001.

To construct a risk score assessment for predicting the OS of patients with MAC, LASSO regression analysis was used based on the aforementioned 71 hub genes. Eventually, a prognostic signature was constructed containing 10 hub genes: TROAP, C19orf48, CCNF, ZMYND19, RUVBL1, PAFAH1B3, ENTR1, NTMT1, RANGAP1, and IFRD2 (**Figures 5A, B**). These ten hub genes, shown in **Figure 5C**, were closely related to each other in the expression of mRNA, which indicated that they may be functionally related. We used Cox proportional hazards regression analysis of the hub genes to develop a prognostic signature. After excluding patients with follow-up periods of less than 30 days, patients with MAC were divided into low-and high-risk groups, according to the risk scores. Clinical characteristics of the high-risk and low-risk groups were shown in **Supplementary Table S3**. There were no significant differences in clinical characteristics between the two groups, except for more patients in the high-risk group with stage N2 (*p* = 0.025). The distribution of risk scores was analyzed, and the relationship between the prognostic signature and the expression of the 10 hub genes was observed. A marked decrease in the OS of patients with MAC was observed as the risk score increased (**Figure 5D**). The Kaplan-Meier survival curve revealed that patients in the high-risk group had significantly worse OS than those in the low-risk group (*p* < 0.0001, hazard ratio = 21.40, 95% CI 2.82–162.61, **Figure 5E**). Hereafter, we explored the performance of the ten hub genes-based signature for prognosis prediction. The AUCs for 1-, 3-, and 5-year OS were 0.87 (95% CI, 1.00–0.71), 0.92 (95% CI, 1.00–0.81), and 0.89 (95% CI, 1.00–0.72), respectively (**Figure 5F**). ROC curve analysis showed that the signature of the 10 genes had good prognostic prediction ability for patients with MAC.

To further visualize the OS probability of patients with MAC, we constructed a nomogram with age, sex, T stage, N stage, M stage, and the 10 hub genes-based prognostic signature. Depending on the nomogram of total points-to-outcome, patients with higher total points were estimated to have worse 1-, 3-, and 5-year OS probabilities (**Figure 5G**). The C-index of the nomogram was 0.80 (95% CI 0.72–0.89, *p* < 0.001). Moreover, the nomogram calibration curves showed promising performance in predicting 1- and 3-year OS probabilities (**Figure 5H**).

### 3.4 Assessment of the clinical significance of the hub genes

**Figure 6:**
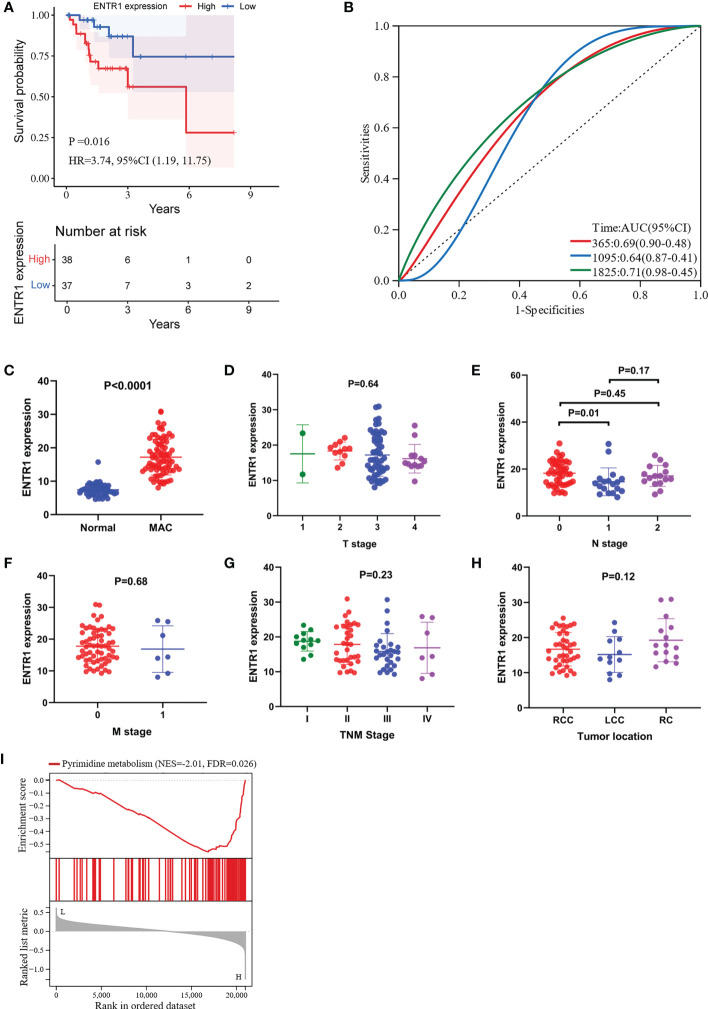
Visualization of the correlation between ENTR1 expression and clinical characteristics. GSEA of ENTR1. **(A)** Kaplan-Meier survival curves for the high-expression group and the low-expression group depending on ENTR1 expression. **(B)** ROC curves for ENTR1. **(C)** Differences in ENTR1 expression between normal and MAC tissues. **(D)** Differences in ENTR1 expression between different T stages. **(E)** Differences in ENTR1 expression between different N stages. **(F)** Differences in ENTR1 expression between different M stages. **(G)** Differences in ENTR1 expression between different TNM grades. T, tumor; N, regional lymph node; M, metastasis. **(H)** Differences in ENTR1 expression between different tumor locations. RCC, right-sided colon cancer. LCC, left-sided colon cancer. RC, rectal cancer. **(I)** GSEA of ENTR1.

When we investigated the prognostic value of the 10 hub genes, only ENTR1 was closely associated with OS. Depending on the expression level of ENTR1, the patients were classified into either a low-expression group (< 50%), or a high-expression group (≥ 50%). Clinical characteristics of the high-risk and low-risk groups were shown in **Supplementary Table S4**. The high-expression group had worse OS than the low-expression group (*p* = 0.016, hazard ratio (HR) = 3.74, 95% confidence interval (CI), 1.19–11.75, **Figure 6A**). The AUCs of ENTR1 for 1-, 3- and 5-year OS were 0.69 (95% CI, 0.90–0.48), 0.64 (95% CI, 0.87–0.41) and 0.71 (95% CI, 0.98–0.45), respectively (**Figure 6B**). We further explored the association between ENTR1 expression levels and clinical features (**Figures 6C-H**). ENTR1 was upregulated in patients with MAC (*p* < 0.0001). However, except that ENTR1 was more highly expressed in stage N0 than in N1 (*p* = 0.01), no differences were found in ENTR1 expression at different T stages, N stages, M stages, TNM stages, or tumor locations.

### 3.5 GSEA for ENTR1

We conducted GSEA to investigate the potential functions of ENTR1 in MAC. GSEA revealed that the ENTR1 expression was associated with pyrimidine metabolism (FDR = 0.026, **Figure 6I**).

### 3.6 Relationship of ENTR1 with cell stemness of MAC and immune infiltration

**Figure 7:**
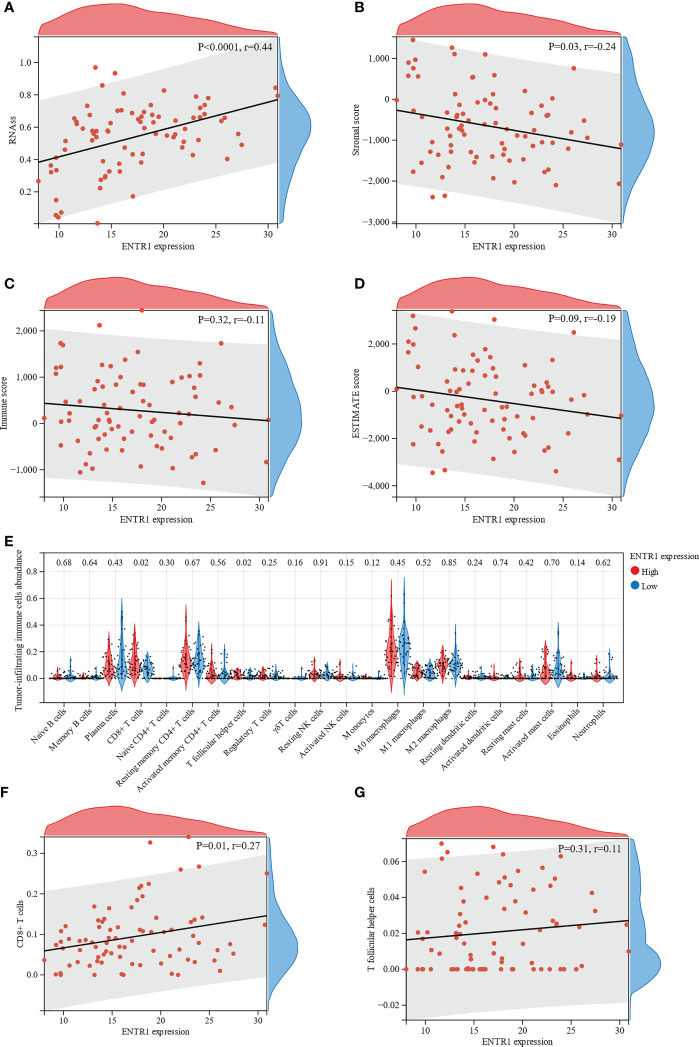
Relationship of ENTR1 with cell stemness of MAC and immune infiltration. Relationship between ENTR1 expression and **(A)** the RNA based Stemness Scores (RNAss), **(B)** stromal scores, **(C)** immune scores, and **(D)** ESTIMATE scores. **(E)** Immune infiltration analysis reveals association of ENTR1 expression and 22 types of tumor-infiltrating immune cells. Relationship between ENTR1 expression and **(F)** CD8+ T cells and **(G)** T follicular helper cells.

To evaluate the correlation between ENTR1 expression and cell stemness of MAC, the RNAss of the MAC samples were calculated using the mRNA signature. The results indicated that ENTR1 expression was significantly positively correlated with the cell stemness of MAC (correlation coefficient = 0.44, *p* < 0.0001, **Figure 7A**).

Subsequently, ESTIMATE was used to assess immune infiltration in the MAC samples. ESTIMATE analysis suggested that ENTR1 expression was negatively correlated with stromal scores (correlation coefficient = -0.24, *p* = 0.03, **Figure 7B**). Immune and ESTIMATE scores displayed a downward trend with increased ENTR1 expression. However, these differences were not statistically significant (**Figures 7C, D**).

The association of ENTR1 with tumor-infiltrating immune cells in the high- and low-expression groups was analyzed using the CIBERSORT algorithm. Analysis of the profile of 22 types of tumor-infiltrating immune cells demonstrated that the number of CD8+ T cells and T follicular helper cells was significantly higher in the high-expression group (*p* = 0.02, **Figure 7E**). Furthermore, we explored the relationship of ENTR1 expression with CD8+ T and T follicular helper cells. The results revealed that ENTR1 expression was positively correlated with CD8+ T cells (correlation coefficient = 0.27, *p* = 0.01, **Figure 7F**), whereas no significant correlation was found with T follicular helper cells (correlation coefficient = 0.11, *p* = 0.31, **Figure 7G**).

### 3.7 Validation of ENTR1 expression

**Figure 8:**
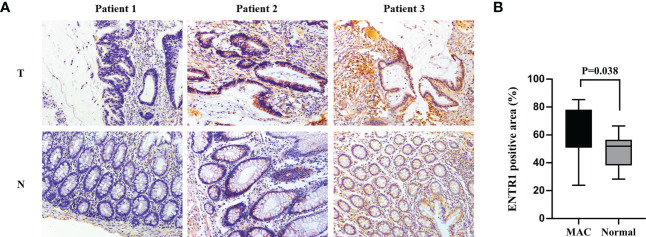
Validation of ENTR1 expression. **(A)** Immunohistochemistry of ENTR1 expression in tumor tissues **(T)** and corresponding normal tissues **(N)** of 3 patients with MAC (x200). **(B)** The proportion of ENTR1 positive area in tumor tissues of MAC and the corresponding normal tissues (immunohistochemistry, n = 13). MAC, mucinous adenocarcinoma.

IHC was used to determine whether ENTR1 expression was higher in the MAC group (n = 13) than in the normal group (n = 13). Ultimately, both groups could be divided into negative (-), low positive (+), and positive (++/+++) groups, based on ENTR1 expression levels. **Figure 8A** showed the extracellular mucus of MAC, and ENTR1 was stained brown and expressed on the cytoplasm and membrane. The overall positive rate of ENTR1 expression in the MAC group was 92.3% (12/13), compared to 69.2% (9/13) in the normal group. The results of the Mann-Whitney-Wilcoxon test showed that there were significant differences in the expression levels of ENTR1 between the two groups (Z = 2.75, *p* = 0.01). The positive area of ENTR1 in the MAC group was significantly higher than that in the normal group (*p* = 0.038, **Figure 8B**).

## 4 Discussion

MAC has been recognized as a separate entity from NMAC, with clear differences in clinical, pathological, and molecular characteristics. Whether the prognosis of MAC is worse than that of NMAC is still controversial, but given that most studies have reported a worse prognosis of MAC (6, 25–28), more prognostic signatures and biomarkers are required to evaluate and predict the prognosis of patients with MAC. To this end, we conducted a comprehensive bioinformatic analysis, using RNA sequencing data from the TCGA database, to construct a solid signature based on hub genes and identify biomarkers for prognosis prediction of patients with MAC.

The identification of DEGs between MAC and normal tissues is a prerequisite for the construction of a reliable and accurate risk score model. We compared the RNA sequencing data between MAC and normal tissues in TCGA database and identified 10,331 DEGs, including ESM1 (29), WNT2 (30, 31), and INHBA (32, 33), which have been reported biomarkers or therapeutic targets for CRC. KEGG and GO enrichment analyses were used to explore the functions of DEGs. The results suggest that KEGG pathways, such as the cAMP signaling pathway and cell adhesion molecules, are linked to MAC pathogenesis. Stachler et al. (34) found that *GNAS* mutations in CRC correlated with the mucinous phenotype, while mutations of GNAS were shown to constitutively activate cAMP signaling (35). Cell adhesion molecules play a key role in peritoneal dissemination (36) and may be associated with increased peritoneal metastasis in MAC. GO analysis indicated that pathways such as the humoral immune response, extracellular matrix, and antigen binding were closely related to MAC development.

WGCNA was used to identify the DEGs that were significantly associated with MAC. The hub genes were then selected from the DEGs in the paleturquoise module, which was most positively associated with MAC. We identified 71 hub genes involved in cell cycle and DNA replication. To construct the optimized risk score model, LASSO-Cox regression analysis was used to further filter the hub genes. Finally, a hub genes-based prognostic signature, including TROAP, C19orf48, CCNF, ZMYND19, RUVBL1, PAFAH1B3, ENTR1, NTMT1, RANGAP1, and IFRD2, was generated. We then measured the risk score of each patient and classified the patients into low-risk and high-risk groups. To validate the performance of this signature, we conducted Kaplan-Meier survival analysis between the two groups and ROC analysis. The Kaplan-Meier survival curve revealed that patients in the low-risk group had a significantly better OS than those in the high-risk group. The AUCs of the time-dependent ROC curves for 1-, 3-, and 5-year OS were 0.87, 0.92, and 0.89, respectively. The Kaplan-Meier survival curve and ROC demonstrated that our prognostic signature could act as a promising predictor of survival in patients with MAC, and aid in clinical decision-making. Furthermore, we constructed a nomogram based on age, sex, T stage, N stage, M stage, and the prognostic signature, to visualize the OS probability of patients with MAC. The C-index and calibration curves showed good predictive performance of the nomogram. However, owing to the limited number of patients with MAC, more data will be required to validate our prognostic signature and nomogram in the future.

Kaplan-Meier survival analysis was performed for all 10 hub genes, and ENTR1 was found correlated with OS. ENTR1, located on chromosome 9, q34.3, was originally identified as an antigen in serum derived from colon cancer patients and was called serologically defined colon cancer antigen 3 (SDCCAG3) with two major splicing variants (37). GSEA and GO enrichment analyses revealed that ENTR1 was associated with pyrimidine metabolism and cell division, respectively. As an endosomal protein localized to early and recycling endosomes, ENTR1 overexpression was found related to an increased number of multinucleate cells, which can lead to chromosome instability and tumorigenesis (38), suggesting that ENTR1 is involved in the regulation of cytokinesis (39), which is in accordance with our results. Liu et al. found that lncHUPC1/miR-133b/SDCCAG3 network could enhance growth and proliferation and reduced apoptosis in prostate cancer (40). Erdmann et al. proposed that ENTR1 binds to Fas through the protein tyrosine phosphatase, PTPN13, and connects to the endosomal sorting complexes required for transport (ESCRT) machinery *via* dysbindin, regulating post-endocytic sorting of Fas, to resist Fas-induced apoptosis (41). At the same time, they also found that depletion of ENTR1 in HCT116, a CRC cell line, could activate caspase 8 and caspase 3 mediated apoptosis and the level of intracellular ENTR1 was regulated by caspases 6 and caspase 8 (41). These may be the reasons for the poor prognosis of patients with high ENTR1 expression.

The tumor immune microenvironment has been identified to play a significant role in the development and progression of CRC, and thus may present potential prognostic factors and therapeutic targets for the disease (42, 43). However, studies related to the tumor immune microenvironment in MAC are scarce. To explore the potential mechanisms of ENTR1 in MAC, we used ESTIMATE and CIBERSORT to assess immune infiltration. Although the results of CIBERSORT showed that ENTR1 expression was positively correlated with CD8+ T cell infiltration, the results of ESTIMATE suggested that ENTR1 expression was negatively associated with stromal scores, and both the immune and ESTIMATE scores also showed a downward trend. In addition, we found that the expression of ENTR1 was positively correlated with the cell stemness. Cancer stem cells (CSCs) are a small subpopulation of cells characterized by embryonic stem cells (ESCs) signatures (44), which can proliferate extensively and drive tumorigenic growth (45, 46). The existence of CSCs in CRC and their significant contributions to clinical tumor progression, chemoradiotherapy resistance, and therapeutic failure have been suggested in several preclinical studies (47–49). This may represent a potential mechanism by which ENTR1 overexpression leads to a poor prognosis in patients with MAC.

Finally, we validated ENTR1 expression in MAC and paired normal colorectal tissues by IHC. The results demonstrated that ENTR1 expression in MAC tissues was higher than that in paired normal tissues, which is in accordance with the finding gained from the TCGA database.

However, our study had some limitations. First, little data are available for MAC because of the low incidence of MAC. And there are only 6 patients with stage M1 out of 75 patients. Therefore, our prognostic signature and nomogram need to be further validated and updated with more appropriate datasets to improve their prognostic ability in the future. Second, we only analyzed the association between expression of ENTR1 and OS without disease-specific survival due to lack of information. Third, although we found that ENTR1 expression is associated with cell stemness, we only validated the expression level of ENTR1. The underlying molecular mechanisms need to be further elucidated through *in vitro* and *in vivo* studies.

## 5 Conclusions

In summary, we established the first prognostic signature, based on WGCNA and LASSO-Cox regression analyses that might be effective in the prediction of prognosis in patients with MAC and further determined that ENTR1 could serve as a prognostic marker for MAC. Our study is promising for the clinical stratification and personalized treatment options for patients with MAC.

## Data availability statement

Publicly available datasets were analyzed in this study. This data can be found here: https://portal.gdc.cancer.gov/.

## Ethics statement

The studies involving human participants were reviewed and approved by the Medical Ethics Committee of Peking University Shougang Hospital. The patients/participants provided their written informed consent to participate in this study.

## Author contributions

Conceptualization, JG. Data curation, JS. Formal analysis, AH. Funding acquisition, JG. Investigation, AH and JS. Methodology, AH. Resources, ZG. Software, AH. Supervision, JG. Validation, JS. Writing – original draft, AH. Writing – review & editing, JS, ZS and YY. All authors contributed to the article and approved the submitted version.
