# Supplementary material for: Identification of a prognostic signature and ENTR1 as a prognostic biomarker for colorectal mucinous adenocarcinoma
Source: Front Oncol. 2023 Apr 27;13:1061785. doi: 10.3389/fonc.2023.1061785 (PMC10172661; doi:10.3389/fonc.2023.1061785)
Supplement: Supplementary file 1 [file DataSheet_1.zip › Table S1.docx]

**Table S1.** Characteristics of the MAC and normal tissues used for analysis.

|  | MAC | Normal tissues | P |
| --- | --- | --- | --- |
| Age |  |  | 0.566 |
| ≥50 | 66 | 44 |  |
| <50 | 14 | 7 |  |
| Sex |  |  | 0.584 |
| Male | 40 | 23 |  |
| Female | 40 | 28 |  |
| Tissue location |  |  | 0.903 |
| Colon | 65 | 41 |  |
| Rectum | 15 | 10 |  |

P value of the χ^2^ test was used to compare the MAC and normal groups. MAC, mucinous adenocarcinoma.
