# Supplementary material for: Identification of a prognostic signature and ENTR1 as a prognostic biomarker for colorectal mucinous adenocarcinoma
Source: Front Oncol. 2023 Apr 27;13:1061785. doi: 10.3389/fonc.2023.1061785 (PMC10172661; doi:10.3389/fonc.2023.1061785)
Supplement: Supplementary file 1 [file DataSheet_1.zip › Table S3.docx]

**Table S3.** Characteristics of the high-risk and low-risk groups.

|  | High-risk group | Low-risk group | P |
| --- | --- | --- | --- |
| Age |  |  | 0.720 |
| ≥50 | 32 | 30 |  |
| <50 | 6 | 7 |  |
| Sex |  |  | 0.907 |
| Male | 19 | 19 |  |
| Female | 19 | 18 |  |
| T stage |  |  | 0.689 |
| T1 | 1 | 1 |  |
| T2 | 7 | 4 |  |
| T3 | 23 | 27 |  |
| T4 | 7 | 5 |  |
| N stage |  |  | 0.025 |
| N0 | 23 | 21 |  |
| N1 | 5 | 13 |  |
| N2 | 10 | 3 |  |
| M stage |  |  | 0.695 |
| M0 | 34 | 35 |  |
| M1 | 4 | 2 |  |
| TNM stage |  |  | 0.756 |
| Ⅰ | 7 | 5 |  |
| Ⅱ | 15 | 16 |  |
| Ⅲ | 12 | 14 |  |
| Ⅳ | 4 | 2 |  |
| Chemotherapy |  |  | 0.933 |
| Yes | 13 | 13 |  |
| No | 25 | 24 |  |

P value of the χ^2^ test was used to compare the high-risk and low-risk groups.
