# Supplementary material for: Identification of a prognostic signature and ENTR1 as a prognostic biomarker for colorectal mucinous adenocarcinoma
Source: Front Oncol. 2023 Apr 27;13:1061785. doi: 10.3389/fonc.2023.1061785 (PMC10172661; doi:10.3389/fonc.2023.1061785)
Supplement: Supplementary file 1 [file DataSheet_1.zip › Table S4.docx]

**Table S4.** Characteristics of the ENTR1 high-expression and low-expression groups.

|  | ENTR1 expression | | P |
| --- | --- | --- | --- |
|  | High | Low |  |
| Age |  |  | 0.720 |
| ≥50 | 32 | 30 |  |
| <50 | 6 | 7 |  |
| Sex |  |  | 0.204 |
| Male | 22 | 16 |  |
| Female | 16 | 21 |  |
| T stage |  |  | 0.131 |
| T1+T2 | 10 | 3 |  |
| T3+T4 | 28 | 34 |  |
| N stage |  |  | 0.004 |
| N0 | 27 | 17 |  |
| N1 | 3 | 15 |  |
| N2 | 8 | 5 |  |
| M stage |  |  | 0.973 |
| M0 | 35 | 34 |  |
| M1 | 3 | 3 |  |
| TNM stage |  |  | 0.049 |
| Ⅰ+Ⅱ | 26 | 17 |  |
| Ⅲ+Ⅳ | 12 | 20 |  |
| Chemotherapy |  |  | 0.292 |
| Yes | 11 | 15 |  |
| No | 27 | 22 |  |

P value of the χ2 test was used to compare the high-expression and low-expression groups.
